# Comparative transcriptome responses of leaf and root tissues to salt stress in wheat strains with different salinity tolerances

**DOI:** 10.3389/fgene.2023.1015599

**Published:** 2023-02-23

**Authors:** Jianfeng Li, Xin Gao, Xunji Chen, Zheru Fan, Yueqiang Zhang, Zhong Wang, Jia Shi, Chunsheng Wang, Hongzhi Zhang, Lihong Wang, Qi Zhao

**Affiliations:** Institute of Nuclear and Biological Technologies, Xinjiang Academy of Agricultural Sciences, Urummqi, China

**Keywords:** salt-tolerant, wheat, transcriptome, WGCNA, alternative splicing, DEGs

## Abstract

**Background:** Salinity stress is a major adverse environmental factor that can limit crop yield and restrict normal land use. The selection of salt-tolerant strains and elucidation of the underlying mechanisms by plant breeding scientists are urgently needed to increase agricultural production in arid and semi-arid regions.

**Results:** In this study, we selected the salt-tolerant wheat (*Triticum aestivum*) strain ST9644 as a model to study differences in expression patterns between salt-tolerant and salt-sensitive strains. High-throughput RNA sequencing resulted in more than 359.10 Gb of clean data from 54 samples, with an average of 6.65 Gb per sample. Compared to the IWGSC reference annotation, we identified 50,096 new genes, 32,923 of which have functional annotations. Comparisons of abundances between salt-tolerant and salt-sensitive strains revealed 3,755, 5,504, and 4,344 genes that were differentially expressed at 0, 6, and 24 h, respectively, in root tissue under salt stress. KEGG pathway analysis of these genes showed that they were enriched for phenylpropanoid biosynthesis (ko00940), cysteine and methionine metabolism (ko00270), and glutathione metabolism (ko00480). We also applied weighted gene co-expression network analysis (WGCNA) analysis to determine the time course of root tissue response to salt stress and found that the acute response lasts >6 h and ends before 12 h. We also identified key alternative splicing factors showing different splicing patterns in salt-sensitive and salt-tolerant strains; however, only few of them were differentially expressed in the two groups.

**Conclusion:** Our results offer a better understanding of wheat salt tolerance and improve wheat breeding.

## Background

Soil salinization is one of the harshest environmental factors and reduces the annual production of various plants. More than 6% of the world’s total agricultural land is affected by high salinity, a proportion that continues to increase (Parihar et al., 2015). Salt accumulation in soils of arid and half-arid regions mainly occurs due to water with trace amounts of NaCl (Tester and Davenport, 2003), which generally impairs the ability of the root to absorb water, leading to the disruption of metabolic processes and reduced photosynthetic efficiency (Maser et al., 2002). However, plants also develop mechanisms to relieve osmotic stress caused by ion cytotoxicity, including decreasing water loss and increasing water uptake (Deinlein et al., 2014). Additionally, many plants reduce Na^+^ ion cytotoxicity by excreting it from their leaves or compartmentalizing it into vacuoles (Blumwald, 2000).

The environment has constantly shaped plant genomes; however, the underlying genetic mechanisms of how plants adapt to environmental influences remain largely unknown. Identifying the corresponding genes and/or mechanisms involved in salinity tolerance provides opportunities to improve crop resistance to soil salinization. Previous studies reported that sulfur (S) assimilation plays an important role in salt stress metabolism. Increased S increased salt tolerance in different plants (Mehar Fatma et al., 2016). Moreover, S-adenosyl methionine (SAM) is the precursor of polyamines (PAs), which also have close relationships with plant resistance to salinity stress (Zhang et al., 2014). Except for these genes, many transcriptional factor (TF) families are involved in response to salt stress, including AP2/ERF, bHLH, bZIP, MYB, NAC, and WRKY(Serra et al., 2013; Yu et al., 2016; Mao et al., 2017). The AP2/ERF superfamily comprises ERF, AP2, RAV, and the Soloist family based on AP2 domains. A total of 26 RAV genes have been identified in wheat (Karami et al., 2022). The NAC family is one of the largest families of plant-specific TFs; its members were derived from three genes containing domains of no apical meristem (NAM), *Arabidopsis* transcription activation factor (ATAF), and cup-shaped cotyledon (CUC) (Aida et al., 1997). NAC1 overexpression in rice led to high salt tolerance (Saad et al., 2013; Chen et al., 2014).

Wheat (*Triticum aestivum*) is one of the most important edible crops globally; however, its yields are seriously threatened by land salinization (Colmer et al., 2006). Wild wheat (*Triticum aestivum* L.) shows potential for improving its raw yield, quality, and tolerance to both biotic and abiotic stresses (Wani et al., 2022). Although wheat currently provides approximately 20% of the world’s caloric intake, significant wheat breeding efforts are needed for this crop to feed the estimated world population of 9 billion by 2050. Using high-throughput RNA-Seq data, many studies have assessed genes involved in wheat salt stress. Zhang Y. et al. (2016) reported that genes including histone-lysine N-methyltransferase, NAC TF, MYB TF, and *TaRSL4* are necessary for salt stress tolerance in the root tissue of bread wheat. Goyal et al. (2016) reported that other genes were responsible for salt tolerance in the root transcriptome of the Kharchia variety, including genes involved in energy supply (like ATP citrate synthase), signaling genes (like Cbl-interacting protein kinase), and ROS scavengers. Xiong et al. (2017) compared expression patterns of shoot between a salt-sensitive wild type and a salt-tolerant mutant bread wheat and found that genes associated with sodium ion transport might be vital for salt tolerance. Moreover, genes encoding arginine decarboxylase, polyamine oxidase, and hormones showed higher expression levels in the salt-tolerant mutant compared to that in the wild type. Amirbakhtiar et al. (2019) compared the transcriptome of root tissue from salt-tolerant bread wheat in Iran, Arg, under salt stress and normal conditions and observed upregulation of genes coding for Ca^2+^ transporters, including *Ta.ANN4*, *Ta.ACA7,* and *Ta.NCL2*.

Previous studies on wheat salt tolerance evaluated single tissues; thus, their results offered limited knowledge about the mechanisms of salt tolerance. In this study, we selected a salt-tolerant wheat strain and conducted a comparative transcriptome analysis of leaf and root tissues. Our results offer a comprehensive understanding of the reactions in salt tolerance.

## Results and discussion

### Samples and sequencing

To better understand the mechanisms underlying acute salt stress of wheat at the transcription level, we used the ST9644 strain. At the seedling stage, samples of almost the same size were chosen and divided into the control and salt-tolerant group. Root samples were collected and sequenced on the Illumina platform at 0, 1, 3, 6, 12, 24, and 48 h, respectively. In total, >1,199.23 million reads were generated, and 359.10 Gb clean reads were kept after data filtering (Supplementary Table S1). The number of reads for each sample ranged from 38 million to 58 million. Among these filtered reads, >92.61% had base quality >Q30 (an error rate of about 0.1%). These values indicated that the quality of filtered data was sufficiently high for the subsequent analyses.

### Mapping statistics for filtering reads

**FIGURE 1:**
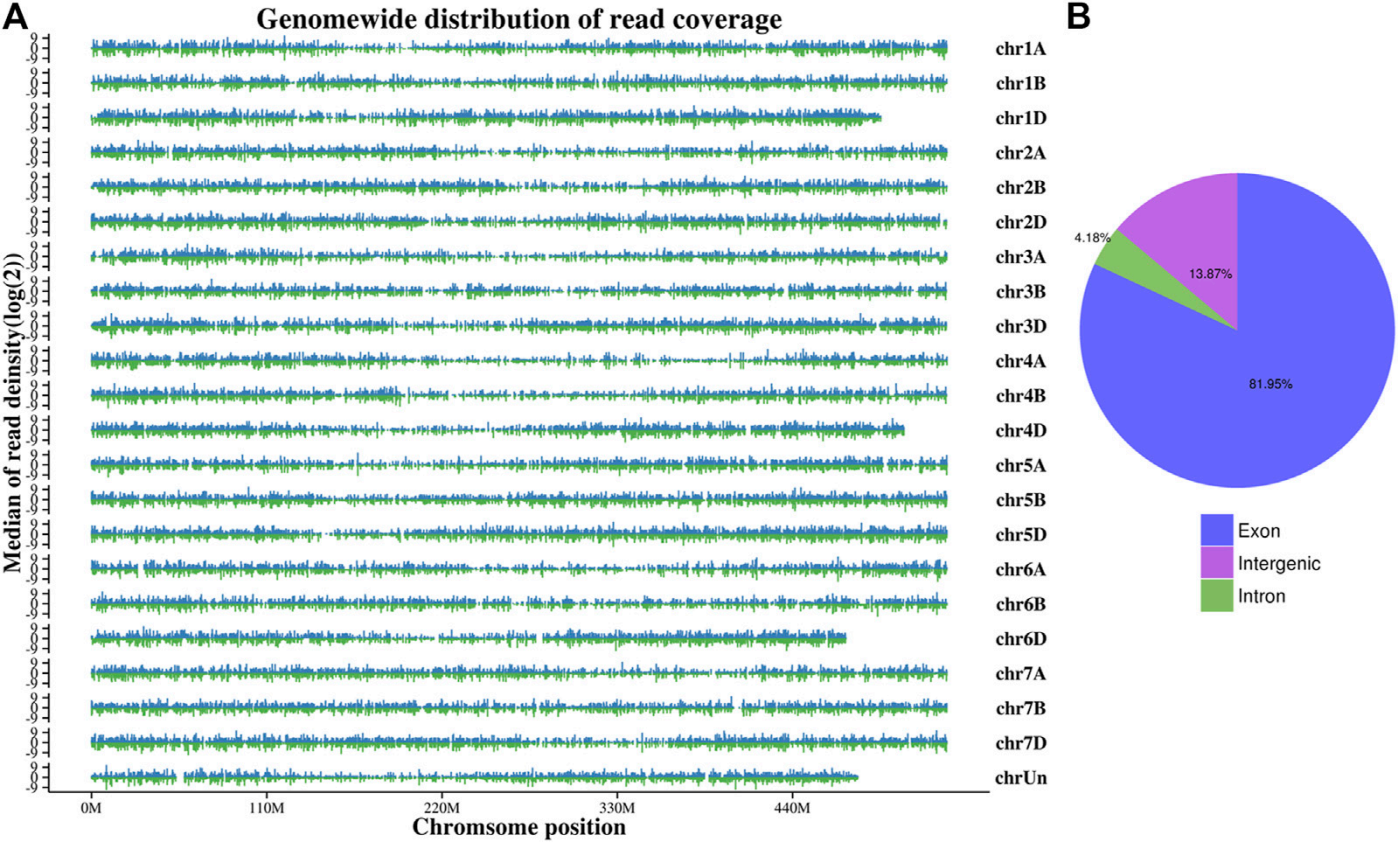
Whole-genome coverage **(A)** and gene read distributions **(B)** for K6G.

Wheat genome-based transcriptome analysis was performed using the Hisat2-Stringtie pipeline (Kim et al., 2019). The alignment results indicated that 75.25%–93.83% of the total reads mapped to the reference genome, among which 71.62%–89.43% were uniquely mapped (Supplementary Table S2). To further assess the quality of libraries, we used whole-genome coverage as an indicator. Our results showed that all libraries demonstrated an even distribution across the wheat genome (Figure 1A). We also visualized the distributions of gene regions for all reads in each sample and found that approximately 82% of reads mapped to the exon region (Figure 1B). With the high-throughput sequencing of the Illumina, we also identified 50,096 new genes in our updated annotation. The annotation results of these new genes showed that 32,923 genes had at least one positive result in annotation databases, including GO, eggNOG, and Swiss-Prot (Table 1).

**TABLE 1:** Functional annotation of all new genes identified in RNA-Seq.

Annotated database	New gene number
GO	14,893
KEGG	4,888
KOG	7,850
Pfam	8,760
Swiss-Prot	9,555
eggNOG	17,640
NR	32,652
All	32,923

### Identification of differentially expressed genes (DEGs)

**FIGURE 2:**
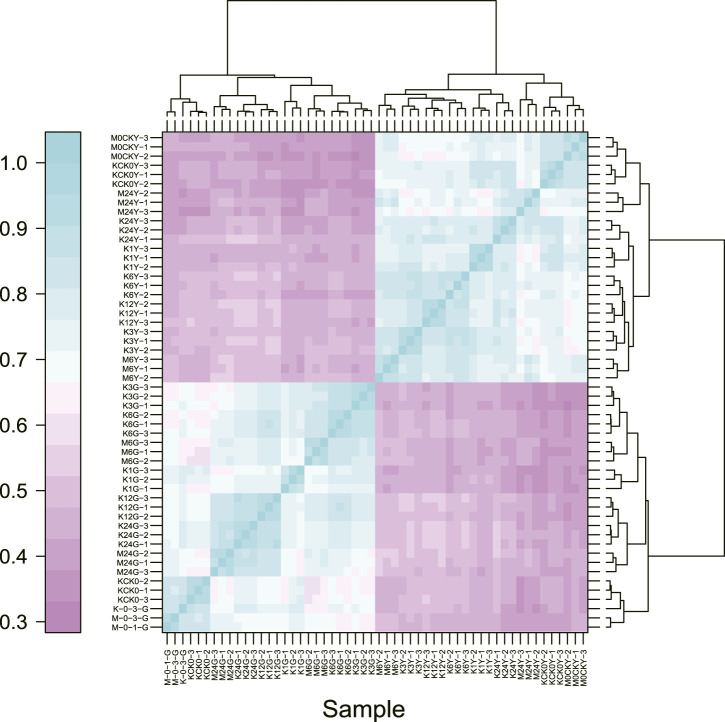
Spearman correlations for all samples based on gene expression.

To determine the sample distances in each tissue, we used Spearman’s correlation coefficients to assess the quality of the RNA-Seq data, which showed that samples from leaves and roots clustered together, as expected (Figure 2). As root tissue is the main organ with direct contact with high salt ion concentrations, we first focused on DEGs at different time points in root tissue between salt-tolerant and salt-sensitive strains. Before the salt stress, we identified 3,755 DEGs between salt-tolerant and salt-sensitive strains, which showed significant enrichment in eight KEGG pathways (Supplementary Table S3; Supplementary Figure S1). Many of these were typical salt-related pathways, including phenylalanine metabolism (ko00360); phenylpropanoid biosynthesis (ko00940); cysteine and methionine metabolism (ko00270); glutathione metabolism (ko00480); and phenylalanine, tyrosine, and tryptophan biosynthesis (ko00400) (Panda et al., 2021). At 6 h after salt stress in root tissue, we identified 5,504 DEGs, including 2,637 upregulated and 2,867 downregulated genes. KEGG enrichment analysis of these DEGs showed significant enrichment of six pathways (Table 2). Among these enriched pathways, phenylpropanoid biosynthesis (ko00940, Supplementary Figure S2) was correlated with salt stress in *Sophora alopecuroides* and barley (Ho et al., 2020; Zhu et al., 2021). Many genes involved in glutathione metabolism and flavonoid biosynthesis were upregulated in the transcriptome of a spaceflight-induced salt-tolerant wheat mutant (Xiong et al., 2017). We also identified many TFs in these DEGs, including NAC, FAR1, MYB, bHLH, bZIP, and WRKY, which were involved in the salt-tolerant strain (Supplementary Figure S3) (Yu et al., 2016; Mao et al., 2017). At 24 h after salt stress in root tissue, we identified 4,344 DEGs between salt-sensitive and salt-tolerant strains, including 1,928 upregulated genes and 2,416 downregulated genes. Six pathways were significantly enriched (Table 3). Among them, glycolysis/gluconeogenesis (ko00010) was stimulated at <50 mM NaCl in the salt-tolerant desert plant *Zygophyllum xanthoxylum* (Chai et al., 2019). Glutathione metabolism (ko00480) and cysteine and methionine metabolism (ko00270) were both involved in eliminating reactive oxygen and were upregulated under salt-tolerant conditions (Xiong et al., 2017; Chai et al., 2019).

**TABLE 2:** KEGG enrichment analysis of 5,504 DEGs in root tissue at 6 h after salt stress.

KEGG_pathway	ko_id	Cluster_frequency	Genome_frequency	*p*-value	Corrected_*p*-value	rich_factor
Phenylpropanoid biosynthesis	ko00940	9.98% (81 out of 811)	5.47% (1122 out of 20488)	1.13E-07	1.36E-05	1.823773
Glutathione metabolism	ko00480	5.17% (42 out of 811)	2.56% (525 out of 20488)	1.33E-05	0.001600831	2.021011
Diterpenoid biosynthesis	ko00904	2.21% (18 out of 811)	0.72% (149 out of 20488)	2.55E-05	0.003059418	3.051862
Plant–pathogen interaction	ko04626	7.76% (63 out of 811)	4.65% (954 out of 20488)	4.89E-05	0.00587346	1.668287
Linoleic acid metabolism	ko00591	1.35% (11 out of 811)	0.37% (77 out of 20488)	2.14E-04	0.025795422	3.608948
Flavonoid biosynthesis	ko00941	2.95% (24 out of 811)	1.34% (276 out of 20488)	2.75E-04	0.033057802	2.196751

**TABLE 3:** Enriched KEGG pathways for DEGs in 24 h root tissue.

Kegg_pathway	ko_id	Cluster frequency	Genome frequency	P-value	Corrected_P-value	Enrich factor
Glutathione metabolism	ko00480	35 out of 575 (6.08%)	525 out of 20488 (2.56%)	2.32E-06	0.00025764	2.3754203
Glycolysis / Gluconeogenesis	ko00010	36 out of 575 (6.26%)	571 out of 20488 (2.78%)	5.88E-06	0.000652701	2.246457
Fructose and mannose metabolism	ko00051	20 out of 575 (3.47%)	265 out of 20488 (1.29%)	6.42E-05	0.007125824	2.689155
Cysteine and methionine metabolism	ko00270	27 out of 575 (4.69%)	433 out of 20488 (2.11%)	0.0001028	0.011414161	2.2218134
Nitrogen metabolism	ko00910	16 out of 575 (2.78%)	192 out of 20488 (0.93%)	0.0001087	0.012060594	2.9692754
alpha-Linolenic acid metabolism	ko00592	17 out of 575 (2.95%)	217 out of 20488 (1.05%)	0.0001426	0.015831325	2.7913925

**FIGURE 3:**
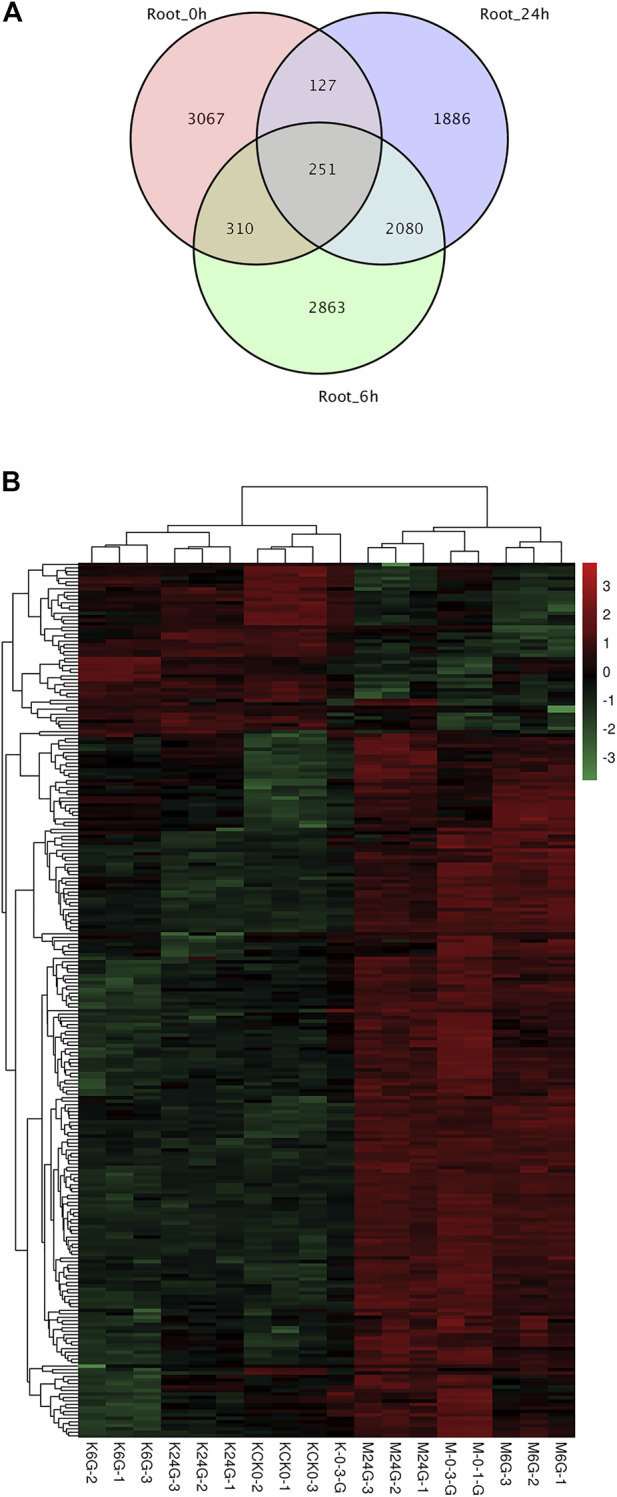
DEG overlap and heatmap at 0, 6, and 24 h in root tissue. **(A)** Overlap of DEGs at three time points. **(B)** Heatmap of 251 DEGs for all three time points.

Among the three time points, we identified 2,331 DEGs with overlap at 6 and 24 h, while few genes overlapped at the other two comparisons (Figure 3A). The reason for this phenomenon might be that salinity caused long-lasting stress to root tissue and reshaped the expression pattern to alleviate the toxicity of high ion concentrations. Among the 2,331 DEGs, 251 were present at all three time points, suggesting their role as housekeeping salt-tolerant proteins. Nine genes were related to phenylpropanoid biosynthesis (ko00940), which showed the largest number of enriched genes (Figure 3B).

**FIGURE 4:**
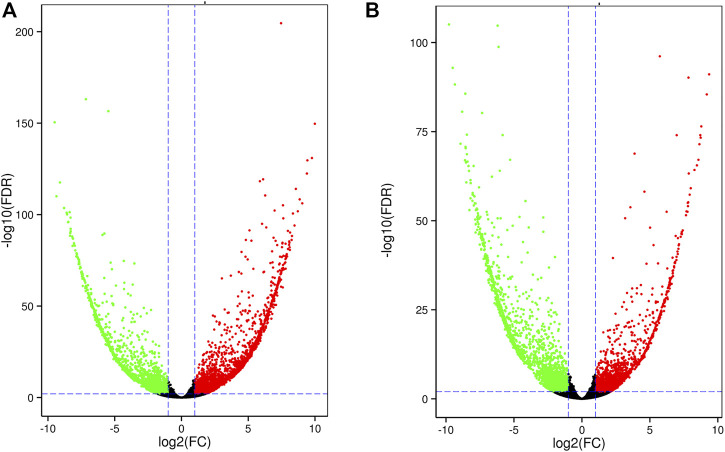
Volcano plot of DEGs in leaf tissue at 6 h **(A)** and 24 h **(B)**.

In leaf tissue, we identified 5,476 DEGs between salt-sensitive and salt-tolerant strains. At 6 h after salt stress, among 4,447 DEGs, 2,546 were upregulated and the rest were downregulated (Figure 4A). KEGG enrichment analysis revealed significant enrichment of three pathways, including phenylalanine metabolism (ko00360), alpha-linolenic acid metabolism (ko00592), and phenylpropanoid biosynthesis (ko00940). Previous studies reported alpha-linolenic acid metabolism among the pathways of salt-responsive proteins in sesame and purslane (Zaman et al., 2019; Zhang et al., 2019). At 24 h after salt stress, among 4,715 DEGs, 2,018 were upregulated and 2,697 were downregulated (Figure 4B). Four KEGG pathways were significantly enriched, including the MAPK signaling pathway (ko04016), plant hormone signal transduction (ko04075), and ribosome (ko03010) and carotenoid (ko00906) biosynthesis. Many different plant hormone signals are responsible for counteracting the toxicity of high ion concentrations (Ryu and Cho, 2015). Among 9,221 DEGs in leaf tissue, 1,639 were observed in all three time points (Supplementary Figure S4). A total of 1,978, 1,272, and 2,193 DEGs were specific to each time point. The overlapping DEGs showed a minor transcription difference compared to root tissue.

### Network analysis of root tissue response to salt stress based on weighted gene co-expression network analysis (WGCNA)

**FIGURE 5:**
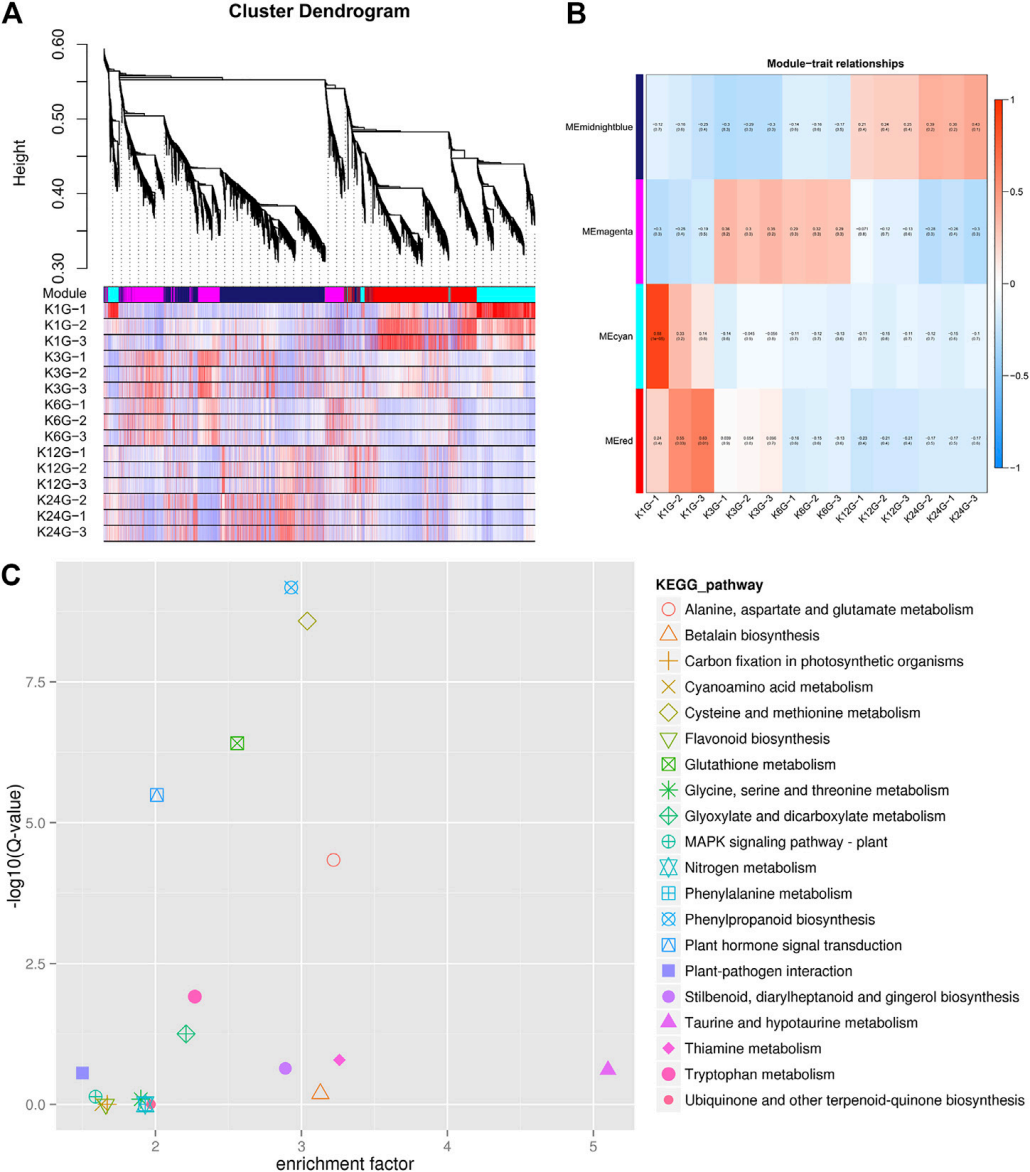
WGCNA analysis of root tissue with salt tolerance. **(A)** Cluster dendrogram of all 15 libraries at five time points in root tissue. **(B)** Module–trait relationships for different modules with salt tolerance. **(C)** KEGG enrichment analysis of the green-yellow module.

To study the expression patterns at different time points in root tissue, we applied WGCNA to identify the relationships between salt stress and differences in gene expression. After merging modules with a minimum height of 0.25 and a minimum module size of 30 genes, we obtained five modules (Figure 5A), which corresponded to six different root stages. The brown module corresponded to the untreated status. KEGG phase analysis showed that phenylpropanoid biosynthesis and cysteine and methionine metabolism were the most highly enriched pathways (Figure 5C). The light yellow module showed the most significantly changed genes 1 h after salt stress. After the acute change, the green-yellow module contained genes appearing within 1 h and 3 h after salt stress. KEGG analysis revealed 23 genes in environmental information processing, 17 of which were involved in plant hormone signal transduction (Figure 5C). We also identified three ABC transporter genes in the pathway. These genes were identified as upregulated DEGs in a previous salt stress experiment in wheat (Amirbakhtiar et al., 2019). The red module showed a similar pattern for genes within 3 and 6 h. Finally, the black module contained genes between 12 and 24 h, which showed no overlap with other stages. This pattern showed that the acute reaction of root tissue to salt stress lasted >6 h. At 12h, the gene expression patterns showed minor differences (Figure 5B).

### Alternative splicing patterns in wheat

**FIGURE 6:**
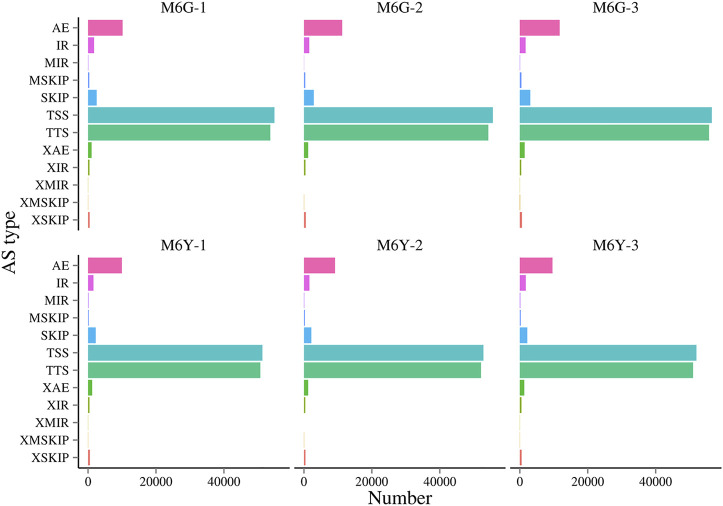
Different alternative splicing types in M6Y and M6G samples.

Alternative splicing (AS) is a common phenomenon in multi-exon eukaryotic genes, through which multiple transcripts can be generated. Many mechanisms have been reported, including mutually exclusive exons (MXEs), alternative exon (AE) ends, skipped exons (SEs), alternative 3′/5′ splicing sites (TTS/TSS), and retained introns (RIs) (Jin et al., 2018). RIs are the most common type of AS in plants and usually result in transcripts with premature termination codons (PTCs), which further lead to non-sense mRNA decay (NMD) (Monteuuis et al., 2019). In this study, root and leaf tissues shared similar patterns of AS at the same time points (Figure 6). Moreover, TSS and TTS were the most common AS types in all the samples, at approximately 40.79% and 40.07%, respectively. However, this pattern is contrary to that in cotton, in which the most common AS under salt stress is intron retention (35.73%) (Zhu et al., 2018). Although the two plants have distinct AS patterns, they both have increased AS events under salt stress in both roots and leaves. This phenomenon may increase broader plasticity for different plants to adapt to various stresses (Zhu et al., 2018).

Previous studies have reported many splicing regulators in plant responses to abiotic stress (Zhan et al., 2015; Zhang F. et al., 2016). The cap-binding protein CBP20 modulated salt stress response in *Arabidopsis* (Kong et al., 2014) and is part of a subunit of dimeric nuclear cap-binding complex (CBC) which combines with the cap structure of RNA polymerase II to influence AS of first introns (Raczynska et al., 2010). In the root tissue in the present study, we identified TSS and TTS at 6 and 24 h in the salt-tolerant strain for *Ta.CBP20*. However, this gene neither show AS in the salt-sensitive strain at the same time point nor was differentially expressed at the two time points. Another large protein family involved in splicing is the serine/arginine (SR) family (Carvalho et al., 2013). Previous studies found that SR overexpression increased plant tolerance to salt or other abiotic stress (Palusa et al., 2007; Duque, 2011; Feng et al., 2015). Moreover, the AS of most SRs was identified under stress conditions (Ding et al., 2014). In this study, we identified 26 SR ortholog genes among wheat annotations. Among them, 10 showed differential alternative splicing under salt stress while only two were differentially expressed under salt stress. This pattern was like that in cotton under salt stress, indicating that splicing regulators in plants preferred to be regulated at the post-transcriptional level rather than the transcriptional level.

### Experimental validation of DEGs by qRT-PCR

**FIGURE 7:**
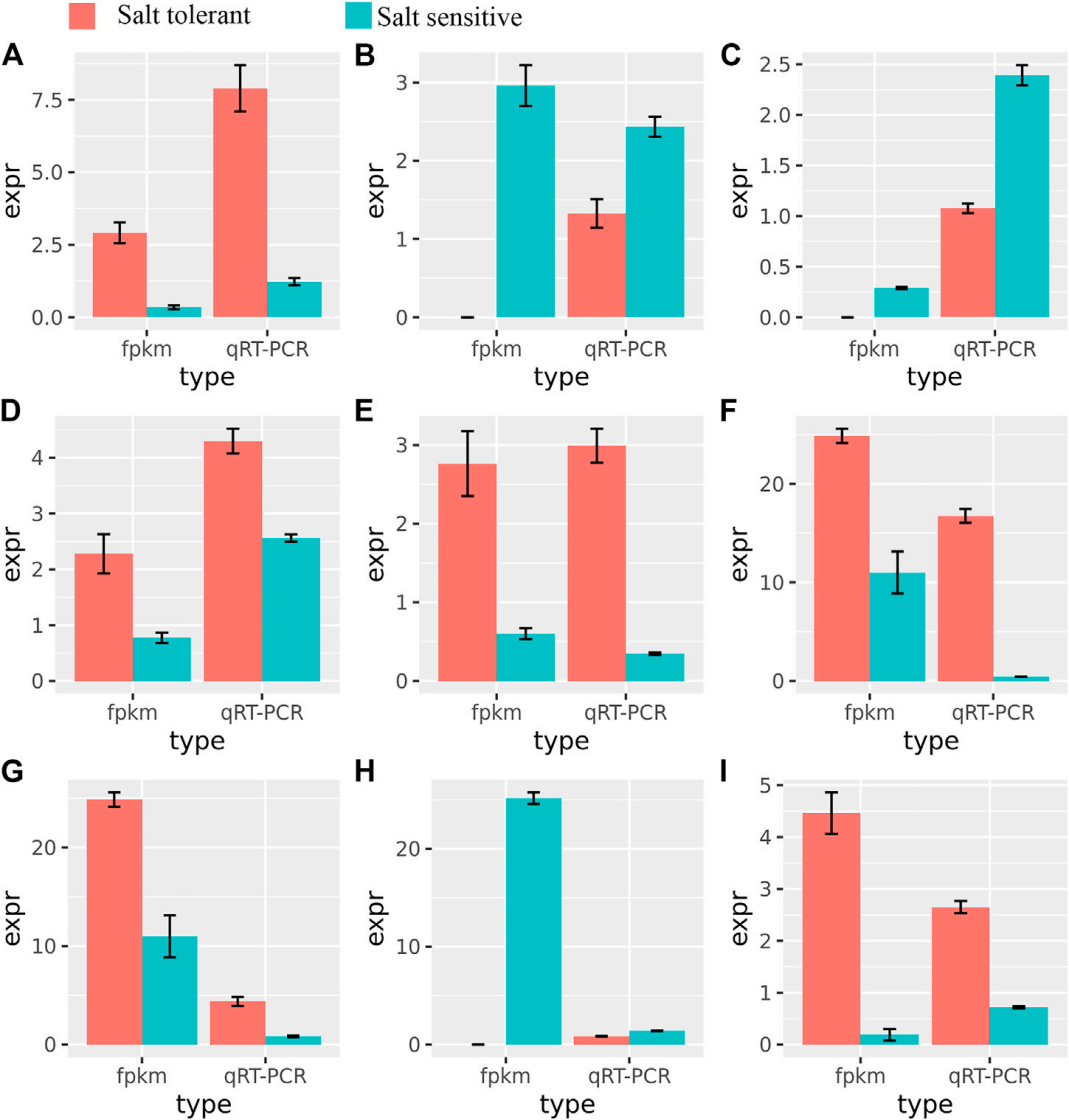
Relationships between FPMK and qRT-PCR validation of nine differentially expressed genes. The expression values (mean ± SE) were used in comparison to those of RNA-Seq. **(A)**: TraesCS6B02G205900; **(B)**: TraesCS5A02G391700; **(C)**: TraesCS5A02G391700; **(D)**: TraesCS4A02G425300; **(E)**: TraesCS5D02G452500; **(F)**: TraesCS2A02G483300; **(G)**: TraesCS5B02G449500; **(H)**: TraesCS2D02G567100; **(I)**: TraesCS6A02G243200.

To validate the RNA-Seq expression at different time points, nine genes related to salt tolerance were selected for further validation by qRT-PCR (Figure 7). The qRT-PCR results were consistent with those from RNA-Seq (R^2^ = 0.96, Supplementary Figure S5), indicating the high accuracy of the DEGs identified in this study. Many of these genes, such as ABC transporters, are reportedly involved in salt tolerance (Amirbakhtiar et al., 2019).

## Conclusion

In this study, high-throughput RNA-Seq of 54 samples from root and leaf tissues in wheat strains with different tolerance on salt stress revealed different expression patterns between the two groups at different time points. Our results showed that the expression pattern of root tissue changed dramatically after salt stress and that the acute reaction lasted for >6 h and ended before 12 h. From that time on, the expression pattern remained relatively consistent for >24 h. KEGG enrichment analysis showed that the DEGs in this study were enriched in phenylpropanoid biosynthesis (ko00940), cysteine and methionine metabolism (ko00270), and glutathione metabolism (ko00480), which have also been identified in other plants under salt stress. Finally, we predicted that different plants shared pathways and mechanisms to cope with salt stress. Our results offer new knowledge for an improved understanding of the mechanisms of salt tolerance in wheat.

## Methods

### Study samples

All samples used in this study were obtained from the 71 strains generated for the Global Challenges Programme (GCP), whose salt tolerances were tested according to the technical specifications for the identification and evaluation of salt tolerance in wheat (NY/PZT001-2002) from the Chinese Ministry of Agriculture. Among these 71 strains, salt-tolerant 9644 (ST9644) showed the best appearance and was chosen as the target in this study. Seeds were placed in a germinating box to the seedling stage and then grown hydroponically in the greenhouse until the two-leaf and one-heart stages. Next, similarly sized plants were divided into the control and salt stress groups. The control group was treated with pure water, while the salt stress group was treated with a 2% NaCl solution. Leaf and root tissues were collected after the treatment begin for 0, 1, 3, 6, 12, 24, and 48 h (Supplementary Figure S6). At each time point, three biological replicates were taken for both groups. All samples were immediately frozen in liquid nitrogen until further analysis.

### Total RNA extraction and Illumina sequencing

Total RNA was extracted from all aforementioned samples using an RNeasy Plant Mini Kit (QIAGEN) according to the manufacturer’s instructions. The RNA purity and concentration were checked using the NanoDrop 2000 and Qubit 2.0 instruments. Samples with RIN values >8.0 were used for library construction and Illumina sequencing. The libraries were constructed using Illumina TruSeq RNA Sample Preparation Kit v2 (Illumina, San Diego, CA, United States) according to the manufacturer’s instructions. Sequencing was performed by Biomarker Ltd. (Beijing, China) on NovaSeq 6000 instruments (Illumina, San Diego, United States) with PE150. To remove low-quality reads or base pairs, reads with adapters, poly-N homopolymers, and very low quality reads were removed from the raw sequencing data. Finally, the Q20 and Q30 values were calculated to further assess the quality of the filtered reads.

### Differentially expressed gene (DEG) identification

Quality-controlled reads were mapped to the wheat genome sequence (https://www.ncbi.nlm.nih.gov/assembly/GCA_900519105.1wgsc_refseqv1.0) by Hisat2 (Kim et al., 2015). Gene expression was quantified as fragments per kilobase of transcript per million (FPKM). Differential expression analysis between the control and salt stress groups was performed using DESeq2, which used a model based on a negative binomial distribution to identify DEGs from the whole gene set (Love et al., 2014). The *p*-values were adjusted using the Benjamini–Hochberg method, and the corresponding false discovery rate (FDR) was determined (Anders and Huber, 2010). Genes with FDR<0.01 and fold-change>2 were assigned as DEGs (Fu et al., 2019). GO and KEGG enrichment analyses were carried out using clusterProfiler (version 3.10.1) (Yu et al., 2012) in R software. Venn graphs of the overlaps of DEGs at different time points were generated using BMKCloud (www.biocloud.net).

### WGCNA analysis

To identify similarities in gene expression patterns among all the samples, we input the log2-normalized FPKM values for all genes into the WGCNA package (Langfelder and Horvath, 2008) in R to generate gene networks. The standard process was used to minimize noise. The gene networks were identified using a dynamic tree-cut algorithm with a minimum cluster size of 25 and merging a threshold of 0.25 (Li et al., 2021). Hub genes were also identified based on eigengene connectivity (KME) (Peter Langfelder and Horvath, 2013).

### Alternative splicing analysis

rMATS was used to identify different AS in transcripts from the same gene in RNA-Seq data (Shen et al., 2014). The statistical model was used to quantify the amounts of alternative splicing events in different samples. The *p*-value was calculated based on the likelihood-ratio test to determine whether these two groups of samples met the inclusion level for which the Benjamini–Hochberg algorithm was used to correct the false discovery rate.

### Quantitative real-time PCR (qRT-PCR) validation

Three replicates for each sample were for qRT-PCR. cDNA was synthesized using a SuperScript IV CellsDirect cDNA Synthesis Kit (ThermoFisher, United States) according to the manufacturer’s instructions. qRT-PCR was performed on a LightCycler 480 Real-time PCR (Roche Life Science, Germany) with the SYBR Green Master Mix (ThermoFisher, United States) according to the manual. Normalization of all genes was performed with TUBB as an internal control. The primers for each gene are listed in Supplementary Table S4. The expression level for each gene was calculated from the cycle threshold using the 2^−ΔΔCT^ method.

## Data Availability

The datasets presented in this study can be found in online repositories. The names of the repository/repositories and accession number(s) can be found in the article/Supplementary Material.

## Supplementary material

SUPPLEMENTARY FIGURE S1 Enriched KEGG analysis of 3,855 DEGs between salt-sensitive and salt-tolerant wheat root tissues at 0 h.

SUPPLEMENTARY FIGURE S2 KEGG pathway for genes enriched in phenylpropanoid biosynthesis (ko00940) between salt-sensitive and salt-tolerant wheat root tissues at 6 h.

SUPPLEMENTARY FIGURE S3 Salt tolerance-related transcription factors in DEGs 6 h after salt stress in root tissue.

SUPPLEMENTARY FIGURE S4 In total, 1,639 DEGs were present in leaf tissue at all three time points (0, 6, and 24 h).

SUPPLEMENTARY FIGURE S5 Correlations between RNA-Seq and qRT-PCR results.

SUPPLEMENTARY FIGURE S6 Workflow of the salt-stressed experiment for the wheat strain ST9644.
