# Supplementary figures and images for: Comparative transcriptome responses of leaf and root tissues to salt stress in wheat strains with different salinity tolerances
Source: Front Genet. 2023 Feb 23;14:1015599. doi: 10.3389/fgene.2023.1015599 (PMC9996022; doi:10.3389/fgene.2023.1015599)

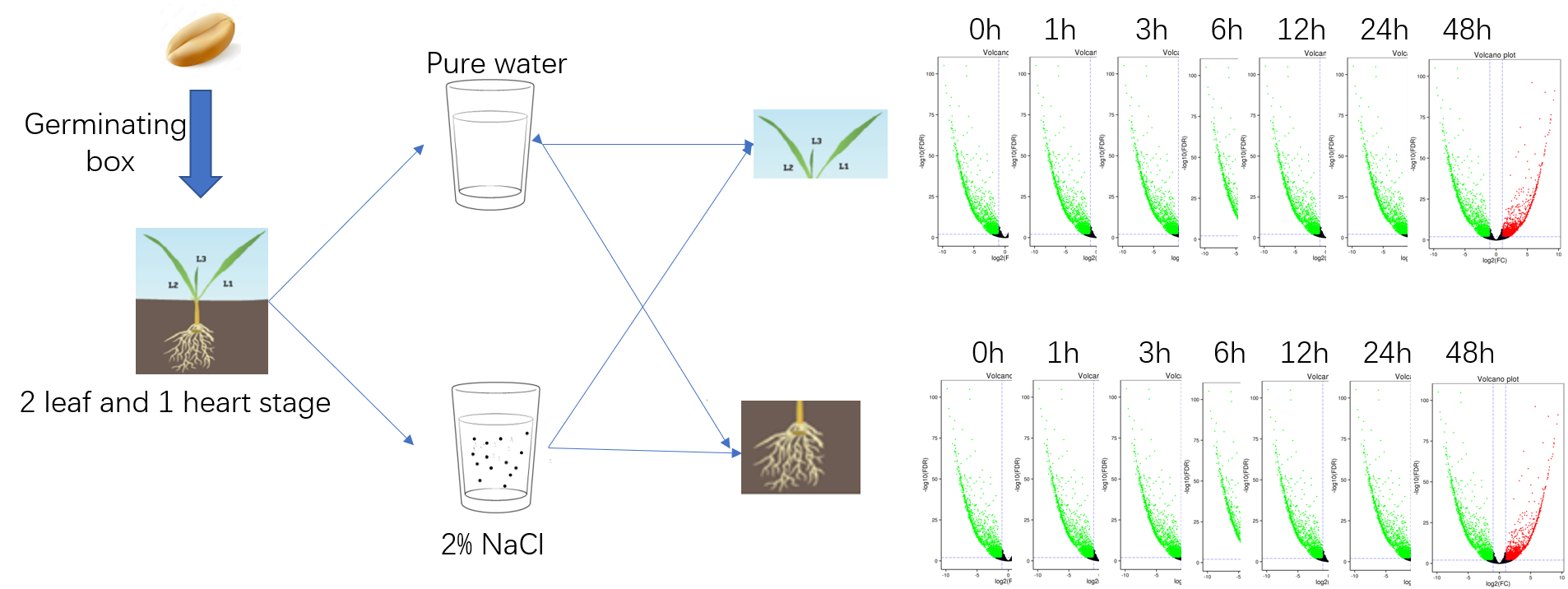

Supplement: Supplementary file 1 [file Image6.TIF]

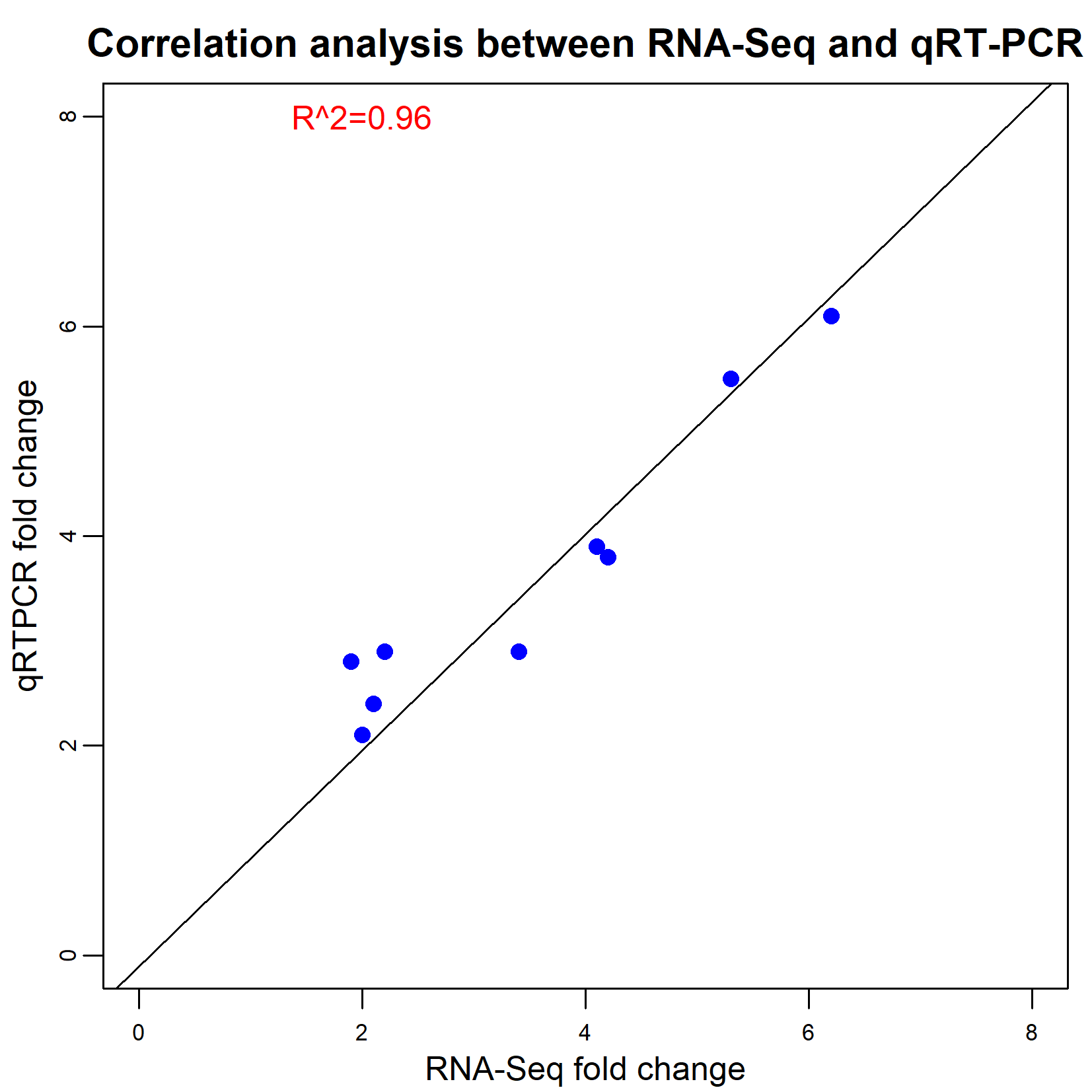

Supplement: Supplementary file 2 [file Image5.TIFF]

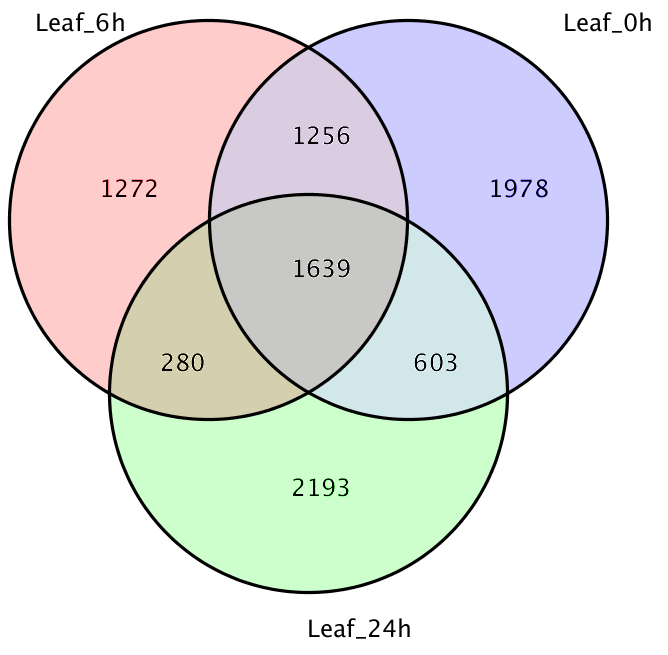

Supplement: Supplementary file 3 [file Image4.PNG]

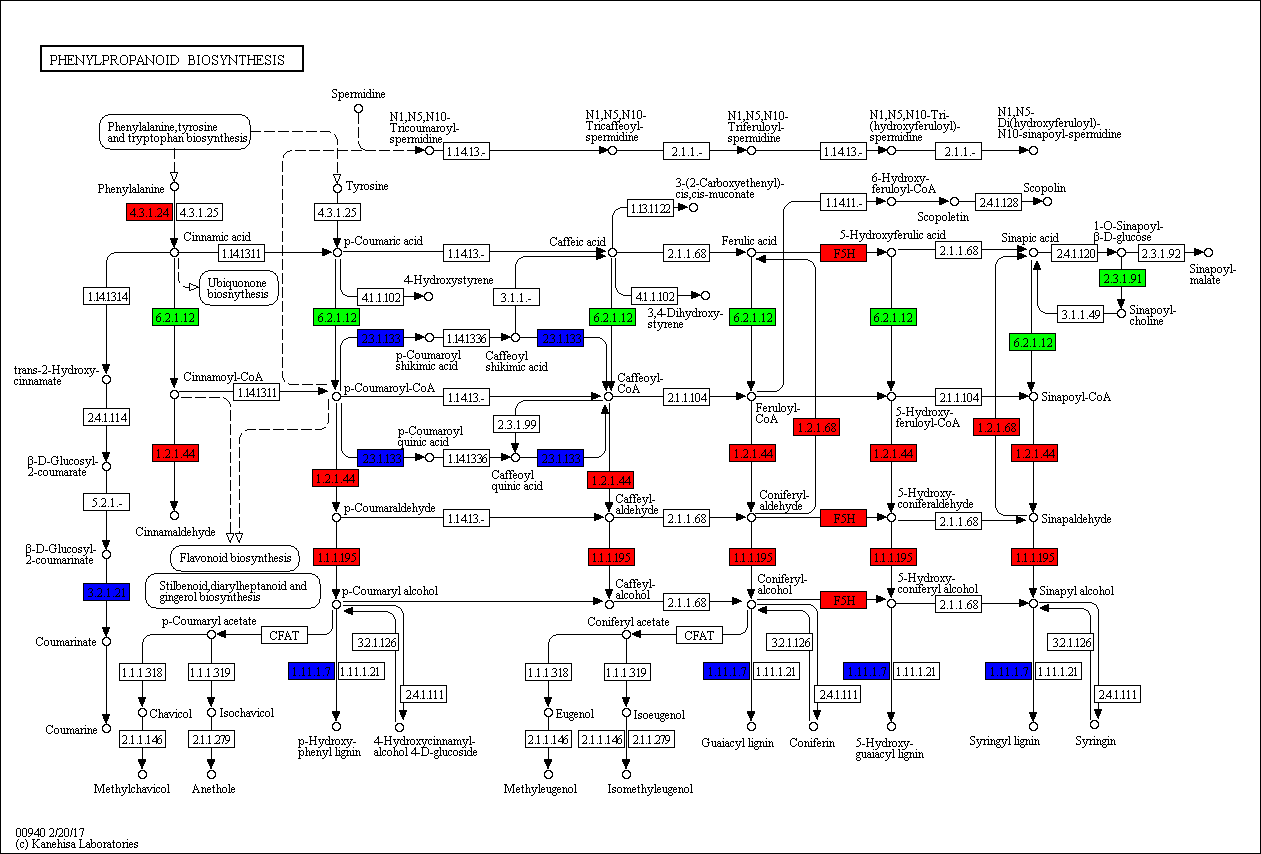

Supplement: Supplementary file 4 [file Image2.PNG]

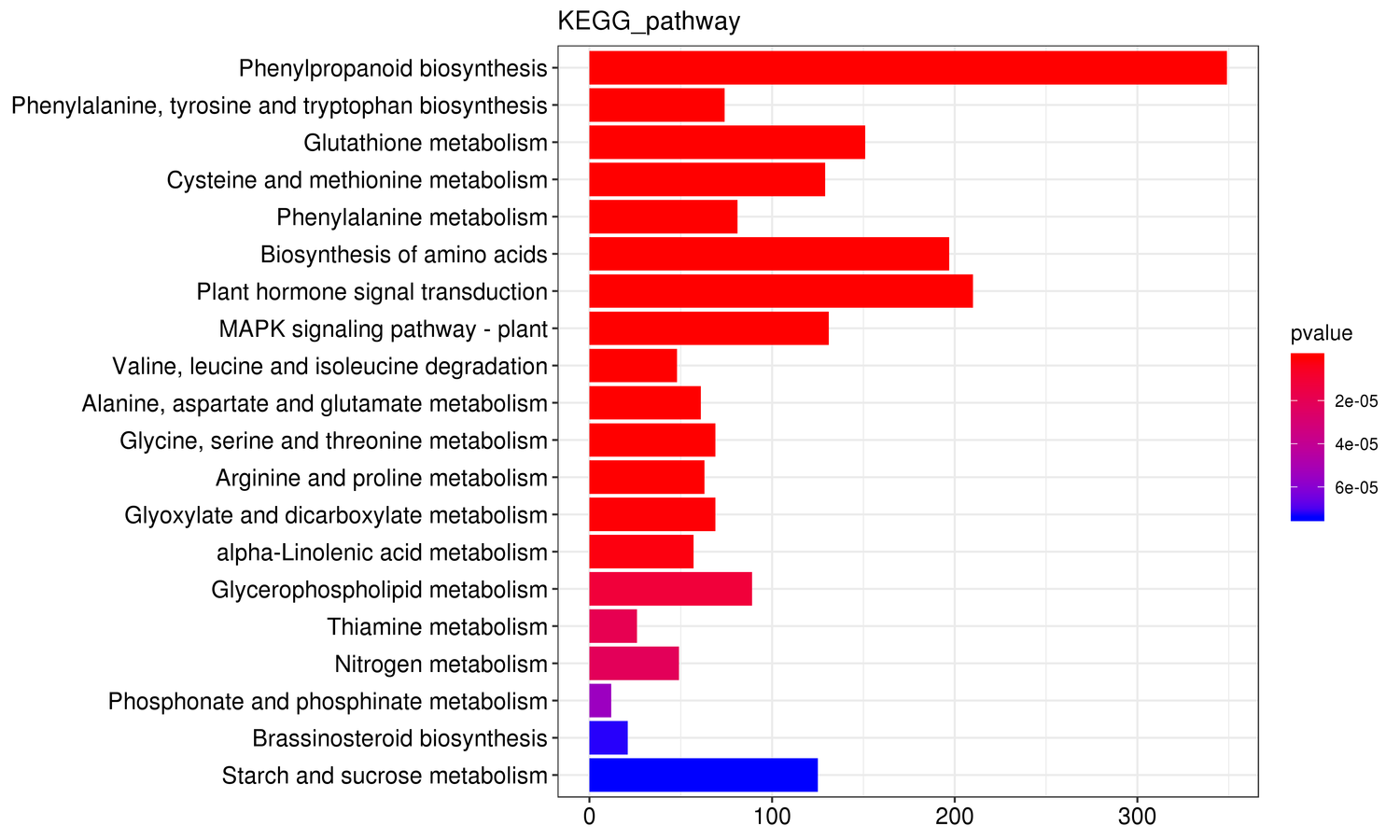

Supplement: Supplementary file 6 [file Image1.PNG]

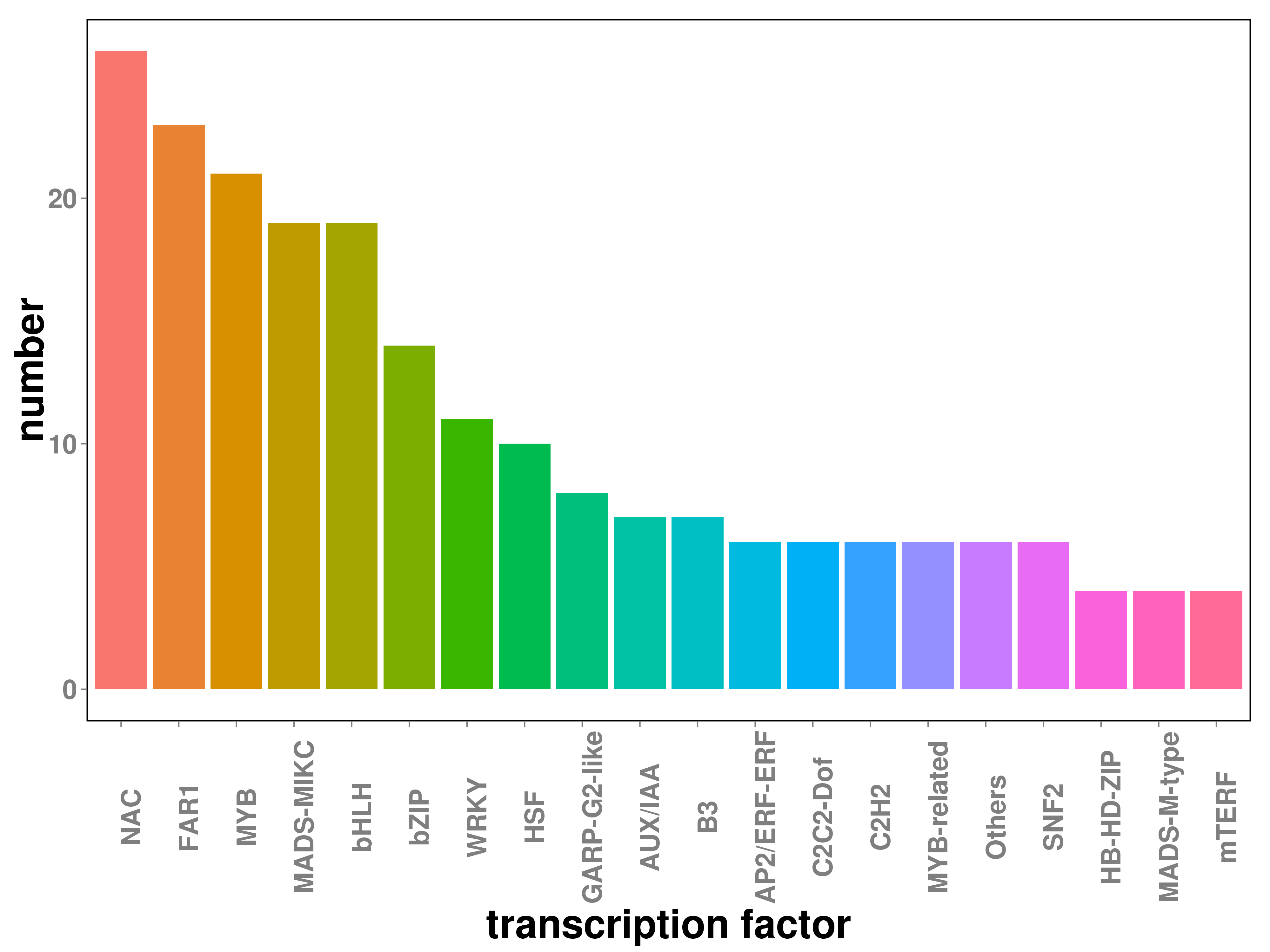

Supplement: Supplementary file 7 [file Image3.PNG]
